# Synchronous Ganglioneuroma and Schwannoma Mistaken for Carotid Body Tumor

**DOI:** 10.1155/2017/7973034

**Published:** 2017-09-24

**Authors:** Konstantinos Paraskevopoulos, Angeliki Cheva, Styliani Papaemmanuil, Konstantinos Vahtsevanos, Konstantinos Antoniades

**Affiliations:** ^1^Department of Oral and Maxillofacial Surgery, General Hospital G. Papanikolaou, 57010 Thessaloniki, Greece; ^2^Department of Pathology, General Hospital G. Papanikolaou, 57010 Thessaloniki, Greece

## Abstract

Ganglioneuromas are a very rare benign neural tumor, commonly derived from the ganglia of the sympathetic system, and are composed of mature Schwann cells, ganglion cells, and nerve fibres. They may arise anywhere from the base of the skull to the pelvis along the paravertebral sympathetic plexus. We report a rare case of synchronous ganglioneuroma and schwannoma, mistaken for carotid body tumor. The coexistence of these two entities in head and neck region is very rare.

## 1. Introduction

Ganglioneuroma (GN) is a very rare entity, one per million population [1]. It is differentiated, benign, neural tumor that commonly derived from the ganglia of the sympathetic system and is composed of mature Schwann cells, ganglion cells, and nerve fibres [2]. Ganglioneuromas may arise anywhere from the base of the skull to the pelvis along the paravertebral sympathetic plexus and occasionally from the adrenal medulla. Common sites of origin are the posterior mediastinum (41.5%), retroperitoneum (37.5%), adrenal gland (21%), and neck (8%) [3, 4].

We present a case of synchronous ganglioneuroma and schwannoma mistaken for carotid body tumor.

## 2. Case Report

A 17-year-old female was referred to Oral and Maxillofacial Department of G. Papanikolaou General Hospital of Thessaloniki, Greece, complaining about a slow-growing neck mass on her left side. She did not complain of any other symptoms. The clinical examination did not reveal any symptoms of cranial nerves. A magnetic resonance imaging (MRI) scan and angiography (MRA) revealed a well surrounded tumor 4,4 × 2,3 × 2,7 cm between the left internal and external carotid artery. It was taken as a carotid body tumor or a paraganglioma. Under general anesthesia, the mass was excised by oral and maxillofacial and vascular surgeons, without any problems during the operation. The postoperative course of the patient was uneventful.

**Figure 1:**
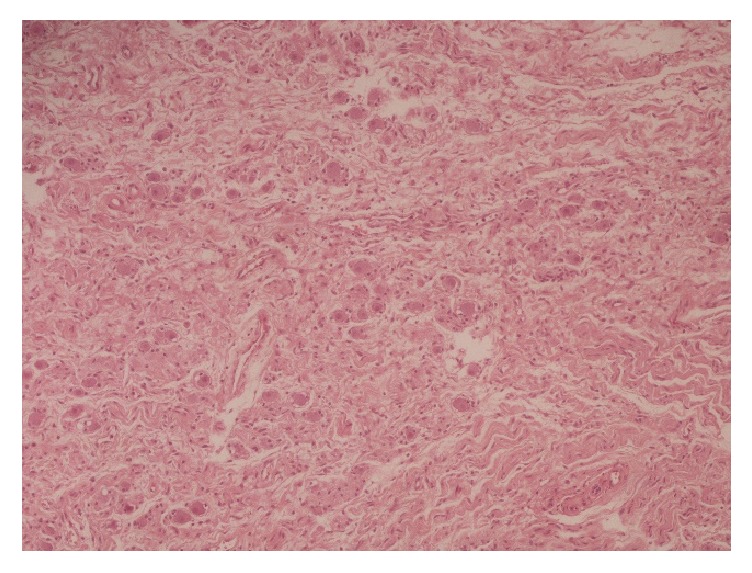
H+E.

**Figure 2:**
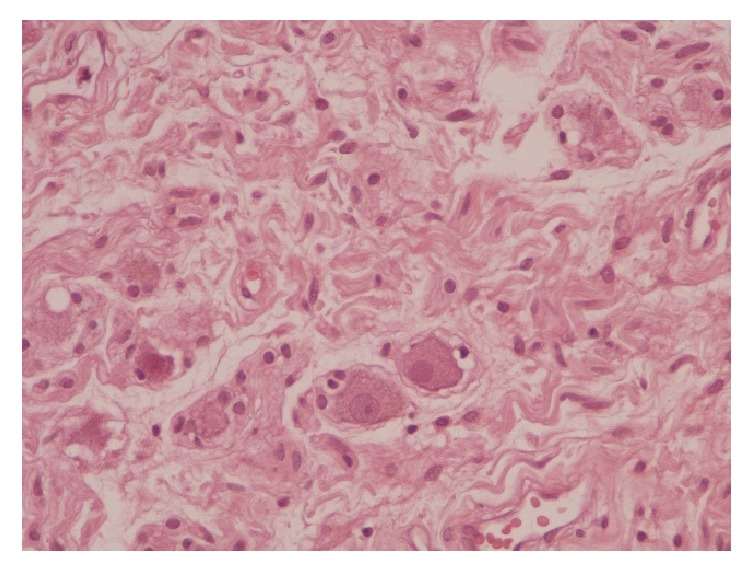
Ganglion cells.

**Figure 3:**
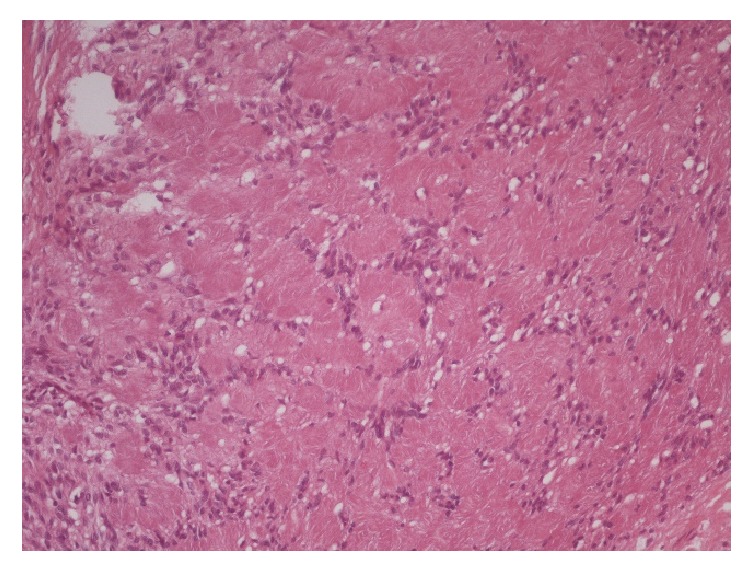
Schwannoma.

The mass was solid, encapsulated, and measured 4 × 2.5 × 1 cm in size. It was whitish in color and soft to firm in consistency. Immunohistochemical analysis showed S-100 (+), GFAP partly (+), Vimentin (+), CD57 (−), Calretinin (−), and AE1/AE2 (−), and a Ki-67 was expressed rarely, which confirmed a diagnosis of schwannoma. It was connected with a nerve which revealed ganglioneuroma with the presence of numerous collections of abnormal but fully mature ganglion cells, often having more than one nucleus (Figures 1–3).

## 3. Discussion

Ganglioneuromas may arise along the paravertebral sympathetic plexus with common sites of origin of the posterior mediastinum (41.5%), retroperitoneum (37.5%), adrenal gland (21%), and neck (8%) [3, 4]. Ganglioneuroma is a slow-growing and well-differentiated, benign tumor which usually develops in childhood (two-thirds under the age of 20 years). They are slow-growing tumors and that is the reason why they are often detected in adults (over the age of 60 years) [5]. Usually these patients do not reveal any clinical symptoms, but they complain of some problems because of compression of surrounding organs. They can cause elevation of the diaphragm, distortion, and subsequent disturbance in functions of the genitourinary or gastrointestinal tract; they can press spinal cord, peripheral nerves, or nerve plexuses and cause distortion and erosion of bony structure [6–10].

In our case, the patient only complained about a slow-growing neck mass on her left side and she did not reveal any other symptoms. Her mass was mistaken for a carotid body tumor and it was treated as it was really a CBT. So after MRI and MRA, it was excised by oral and maxillofacial and vascular surgeons, without any problems during the operation. Pathological examination showed a synchronous ganglioneuroma and schwannoma and not a carotid body tumor. Although the percentage of a neck GN is 8% [3, 4], the coexistence with schwannoma seems to be rare, as there are few mentions in the literature in head and neck region. The treatment of this entity must be the surgical excision to avoid local symptoms because of pressure of close anatomic structures.
